# CD147 promotes breast cancer migration and invasion by inducing epithelial-mesenchymal transition via the MAPK/ERK signaling pathway

**DOI:** 10.1186/s12885-023-11724-2

**Published:** 2023-12-08

**Authors:** Fang Li, Jing Wang, Yu-qiong Yan, Chong-zhi Bai, Ji-qiang Guo

**Affiliations:** 1 Department of Scientific Research, Shanxi Bethune Hospital, Shanxi Academy of Medical Sciences, Third Hospital of Shanxi Medical University, Tongji Shanxi Hospital, Taiyuan, 030032 PR China; 2 Department of Neurosurgery, Shanxi Bethune Hospital, Shanxi Academy of Medical Sciences, Third Hospital of Shanxi Medical University, Tongji Shanxi Hospital, Taiyuan, 030032 PR China; 3 Key Laboratory of Chemical Biology and Molecular Engineering of Ministry of Education, Institute of Biotechnology, Shanxi University, Taiyuan, 030006 PR China; 4 Central Laboratory, Shanxi Province Hospital of Traditional Chinese Medicine, Taiyuan, 030012 PR China; 5 Department of Clinical Laboratory, Shanxi Bethune Hospital, Shanxi Academy of Medical Sciences, Third Hospital of Shanxi Medical University, Tongji Shanxi Hospital, Taiyuan, 030032 PR China

**Keywords:** CD147, Breast cancer, Drug resistance, Epithelial-mesenchymal transitions (EMT), MAPK/ERK signaling pathway

## Abstract

**Background:**

CD147, a transmembrane glycoprotein, has been implicated in various cancer-related processes but its role in breast cancer remains poorly understood. Herein, we investigated the expression of CD147 in different breast cancer cell lines and explored its functional roles, including migration, invasion, drug resistance and modulation of key proteins associated with cancer progression.

**Methods:**

The expression of CD147 was assessed in MCF-10 A, BT549, MDA-MB-231 and MCF-7 breast cancer cell lines using qRT-PCR and Western blotting, following which lyposome transfections were performed, leading overexpression of CD147 in BT549 cells and knockdown of CD147 in MCF-7 cells. Scratch assays and Transwell invasion and were performed to evaluate the cells’ migration and invasion abilities. Sensitivity to 5-FU was determined via CCK-8 assays, and the expression of Snail1, E-cadherin, Vimentin, MMP-9 and the MAPK/ERK pathway were analyzed by qRT-PCR and Western blotting.

**Results:**

Compared with normal beast epithelial cells, CD147 was highly expressed in all breast cancer cell lines, with the highest overexpression observed in MCF-7 cells and the lowest overexpression observed in BT549 cells. Overexpression of CD147 in BT549 cells increased, migration, invasion, viability and resistance to 5-FU of BT549 cells, while CD147 knockdown in MCF-7 cells reduced these properties of MCF-7 cells. Furthermore, CD147 influenced the expression of Snail1, Vimentin, E-cadherin, and MMP-9, suggesting its involvement in epithelial-mesenchymal transition (EMT) regulation. The MAPK/ERK pathway was activated by CD147 in BT549 cells, as indicated by increased p-MEK/MEK ratio and p-ERK/ERK ratio. In contrast, CD147 silencing in MCF-7 cells resulted in reduced p-MEK/MEK ratio and p-ERK/ERK ratio.

**Conclusion:**

In summary, our findings suggest CD147 as a potential therapeutic target in breast cancer treatment, particularly in cases where drug resistance and metastasis are concerns, worthy of further explorations.

**Supplementary Information:**

The online version contains supplementary material available at 10.1186/s12885-023-11724-2.

## Introduction

Breast cancer is the most common cause of cancer-related deaths among women worldwide [1]. The incidence of breast cancer has increased steadily in the last few decades [2], with approximately 1.6 million new cases reported in China in 2020 [3]. Although advances in diagnosis and therapeutic strategies have increased the survival rate of breast cancer patients in recent decades, the prognosis of advanced-stage breast cancer remains dismal due to high recurrence rates and drug resistance [4, 5]. Furthermore, the current treatments have poor efficacy in breast cancer patients with distant metastasis. Therefore, there is an urgent need to identify new and effective therapeutic markers against metastatic breast cancer.

Epithelial-mesenchymal transition (EMT) is a process wherein the epithelial cells lose their polarity and intercellular junctions and acquire the characteristics of the highly motile mesenchymal cells [6]. EMT increases the migratory capacity of epithelial cells, which is crucial for cancer invasion and metastasis, immunosuppression, tissue healing and organ fibrosis [7]. Studies have shown that lung cancer cells undergo invasion and distant metastasis through EMT [8]. The MAPK/ERK signaling pathway regulates several cellular processes, and it has been reported that the phosphorylation of ERK triggers an intracellular signaling cascade that culminates in the activation of transcription factors and protein kinases that regulate pro-survival and cell cycle genes. Further, activation of the MAPK/ERK pathway initiates cell proliferation and inhibits apoptosis [9]. Aberrant phosphorylation of ERK is closely associated with the malignant transformation of cells [10], and the MAPK/ERK pathway is frequently dysregulated during the initiation and metastasis of neurological and urinary system tumors [11]. However, further investigations are required to clarify the exact role of the MAPK/ERK pathway in breast cancer invasion and metastasis, especially considering tumor heterogeneity and different responses of patients despite being classified in the same tumor-node-metastasis (TNM) stage [12].

CD147 is a newly discovered cell surface adhesion factor, which is involved in cell-cell and cell-matrix adhesion [13]. It regulates nutrient transport, inflammatory leukocyte migration, inducing MMPs and VEGF production, spermatogenesis, lymphocyte activation, body immunity, tissue repair, nerve conduction activity, monocarboxylate transporter expression, and production of gamma-secretase complex by amyloid beta peptide in Alzheimer’s disease [14]. Furthermore, CD147 is highly expressed in glioma and liver cancer cells [15, 16] and plays a role in tumor occurrence, development, infiltration and metastasis. The high levels of CD147 on tumor cells induce the production of MMPs and vascular endothelial growth factor (VEGF) from both tumor cells and fibroblasts, promoting the degradation of extracellular matrix and tumor neo-angiogenesis, thereby accelerating tumor cell invasion. Su et al. [17] found that silencing CD147 in melanoma cells reduced VEGF production and inhibited their infiltration and metastasis in vitro by down-regulating the glycolytic pathway. Consistent with this, CD147 stimulated tumor angiogenesis, thereby promoting tumor growth, invasion and metastasis [18]. Altering the expression level of CD147 in human breast cancer cells also affected that of VEGF mRNA and protein [19]. In addition, CD147 regulated the expression of VEGF and VEGF-R2 in melanoma cells through the HIF-2α pathway, thereby promoting angiogenesis [20]. Although previous studies have shown that CD147 is upregulated in breast cancer and could lead to drug resistance and tumor invasion [21], its impact on common chemotherapeutic drugs and the underlying mechanisms remains unclear.

In this study, we aimed to determine whether CD147 regulates EMT of breast cancer cells, its effect on a commonly used chemotherapeutic drugs in clinics and to explore the role of the MARK/ERK signaling pathway in CD147-induced breast cancer invasion and metastasis.

## Materials and methods

### Cell culture

The non-tumorigenic breast epithelial cell line MCF-10 A, the human breast cancer cell lines MCF-7, BT549, and MDA-MB-231 were purchased from ATCC and expanded in DMEM or RPMI 1640 (Gibco, California, USA) supplemented with fetal calf serum (FCS; Institute of Hematology, Hang Zhou, China) and 1% Penicillin-Streptomycin (Gibco, California, USA) within a 5% CO2 incubator. The confluent cultures were homogenized using trypsin (HyClone, Los Angeles, USA). Cells from passages 3 to 6 were used in the experiments.

### Cell transfection

CD147 siRNA (SiCD147) and CD147 cDNA (cCD147) plasmid constructs were purchased from Sangon Biotech. The cells were seeded into 6-well plates at the density of 2 × 10^5^ cells/well and cultured for 24 h till they were 70–90% confluent. The cells were transfected with 2 µg plasmids using Lipofectamine™ 2000 (Invitrogen, USA), and selected 48 h later with 400 µg/ml G418 (Sigma, USA). Stable BT549, BT549-liposome, BT549-liposome-vector, BT549-liposome-CD147, MCF-7, MCF-7-liposome, MCF-7-liposome-siNC and MCF-7-liposome-si-CD147 clones were obtained after 1 month of continuous selection.

### Quantitative real-time polymerase chain reaction (qRT-PCR)

Briefly, Total RNA was extracted from the transfected cells using Trizol (Invitrogen) and reverse transcribed to cDNA using a reverse transcription kit (Takara, Japan). qRT-PCR was performed using SYBR Premix Ex Taq Kit (Takara, Japan) with β-actin as the endogenous control for normalizing gene expression. The primers for CD147 were purchased from Sangon Biotech (Shanghai, China). The forward and reverse primers for CD147 were 5’-CAGCGGTTGGAGGTTGT-3’ and 5’-TTTGAGGGTGGAGGTGG-3’, for β-actin were 5’-AAAGACCTGTACGCCAACAC-3’ and 5’-GTCA TACTCCTGCTTGCTGAT-3’. The PCR cycling conditions were as follows: initial denaturation at 95 °C for 30 s, followed by 40 cycles at 95 °C for 30 s, 60 °C for 30 s, and 72 °C for 30 s. Relative mRNA expression levels were determined by the 2^-ΔΔCt^ method and expressed relative to control cells.

### Western blotting (WB)

Here, WB was performed to assess protein expression levels of the cell groups. Briefly, 1 × 10^6^ harvested cells were lysed with ice-cold RIPA buffer for 30 min, and the protein concentration was determined using the BCA method. Equal amounts of protein per sample were separated by 15% SDS-PAGE and transferred onto PVDF membranes. After blocking with 5% bovine serum albumin in Tris-buffer saline (TBST) at 37 °C for 1 h, the membranes were incubated overnight with primary antibodies (Rabbit anti-human CD147, p-ERK, ERK, β-actin, MMP-9, Snail1, E-cadherin and Vimentin antibodies, Abcam, USA) at 4 °C, and thereafter with an HRP-conjugated secondary antibody for 1 h at room temperature. The protein bands were detected using an enhanced chemiluminescence (ECL) detection kit (Beyotime). The density of each band was normalized to β-actin. Fluorescence intensity was quantified via ImageJ software.

### Cell counting Kit-8 (CCK-8) assay

The resistance of the different cell lines to 5-FU was determined by the Cell Counting Kit-8 (CCK-8) assay according to the manufacturer’s instructions (Beyotime, Shanghai, China). The cells were seeded in 96-well plates at the density of 1 × 10^5^ cells/well in 100 µl medium and cultured for 24 h. After treating the cells with different concentrations of 5-FU (0, 2, 4, 6, 8, 10 µM) for 48 h, 10 µl CCK-8 reagent was added to each well, and the cells were incubated for 2 h at 37ºC. The absorbance was measured at 450 nm using a microplate reader. The dose-response curves were generated to determine their corresponding IC50 values, representing the 5-FU concentration at which cell viability was reduced by 50% compared to untreated controls.

### Scratch wound healing assay

First, the cells were separately seeded and allowed to form a confluent monolayer. Then, a uniform scratch was created across the cell layer using a sterile pipette tip. The cells were then gently washed to remove detached cells and debris, and fresh medium was added. Images of the scratch area were captured at multiple time points post-scratch (e.g., 0, 24 and 48 h). The rate of scratch closure, indicative of cell migration, was quantified by measuring the reduction in scratch width over time to assess the migratory capacity of the different cell groups and the potential impact of CD147 modulation on cell motility.

### Transwell invasion assay

In brief, Transwell inserts with Matrigel-coated membranes were placed into wells containing serum-free culture medium, while cells suspended in serum-free medium were added to the upper chamber. The lower chamber contained a culture medium supplemented with a attractant. After incubation, non-invading cells were removed from the upper surface, and invading cells that passed through the Matrigel and membrane were fixed and stained, then counted under a microscope to quantify the invasive potential of each cell group to determine their invasive capacity.

### Statistical analysis

Statistical analyses were conducted using SPSS 13.0. The results were expressed as mean ± standard deviation (SD) for continuous variables. Statistical significance was assessed using one-way analysis of variance (ANOVA) followed by post hoc tests such as Tukey’s multiple comparison test for multiple groups. P < 0.05 was considered statistically significant.

## Results

### CD147 is highly expressed in breast cancer cell lines

**Fig. 1:**
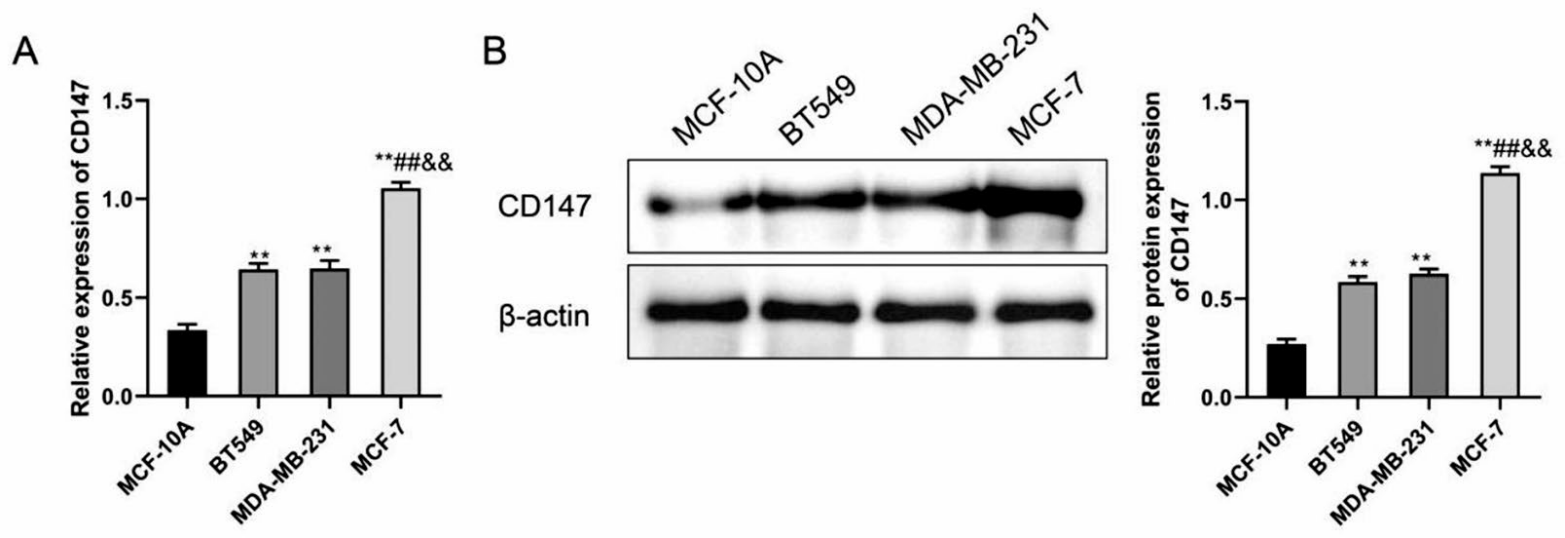
The expression of CD147 in different breast cancer cell lines. (**A**) qRT-PCR analysis of CD147 mRNA levels in MCF-10 A, BT549, MDA-MB-231, and MCF-7 cells. (**B**) Western blot analysis of CD147 protein expression in these cell lines, showing substantial upregulation in BT549, MDA-MB-231, and MCF-7 cells relative to MCF-10 A. **P < 0.01, vs. MCF-10 A; ##P < 0.01, vs. BT549; &&P < 0.01, vs. MDA-MB-231

We assessed the expression levels of CD147 mRNA and protein in the MCF-10 A, BT549, MDA-MB-231 and MCF-7 human breast cancer cell lines via qRT-PCR. Comparative analysis revealed that when compared to the MCF-10 A cell group, the BT549, MDA-MB-231, and MCF-7 cell groups exhibited a significant increase in CD147 gene levels (P < 0.01). When compared to the BT549 and MDA-MB-231 cell groups, the MCF-7 cell group demonstrated a significant elevation in CD147 expression (P < 0.01) (Fig. 1A). Besides, the results of WB analysis were consistent with qRT-PCR results (Fig. 1B). BT549 cells and MCF-7 cells were chose for subsequent experiments.

### Modulating CD147 expression in breast cancer cell lines

**Fig. 2:**
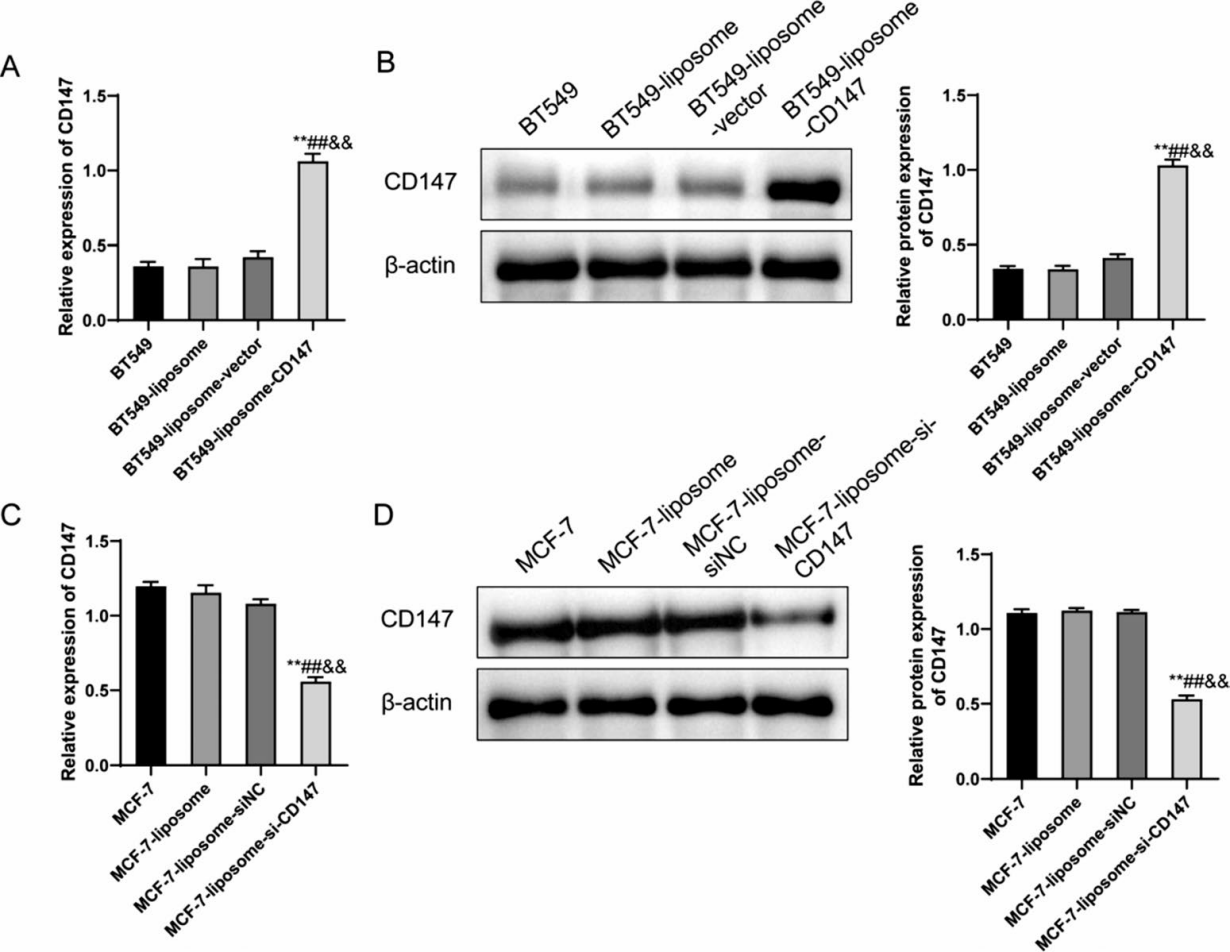
Transfection efficiency of the different constructs in BT549 and MCF-7 cells. (**A**) qRT-PCR results show consistent CD147 expression in BT549, BT549-liposome, and BT549-liposome-vector cells, with successful upregulation in BT549-liposome-CD147. (**B**) Western blot confirms increased CD147 protein expression in BT549-liposome-CD147. (**C**) qRT-PCR demonstrates CD147 knockdown in MCF-7-liposome-si-CD147. (**D**) Western blot confirms reduced CD147 protein expression in MCF-7-liposome-si-CD147. **P < 0.01, vs. BT549/MCF-7; ##P < 0.01, vs. BT549-liposome/MCF-7-liposome; &&P < 0.01, vs. BT549-liposome-CD147/MCF-7-liposome-siNC

Considering that there was a significantly greater expression of CD147 in the MCF-7 cell group than in the BT549 group, further experiments were performed to overexpress the CD147 levels in BT549 cells and knockdown CD147 expression levels in the MCF-7 cells. The qRT-PCR results indicated that CD147 levels remained relatively consistent across BT549, BT549-liposome and BT549-liposome-vector cell groups, with no significant differences observed (P > 0.05), and a successful upregulation of CD147 in the BT549-liposome-CD147 group compared to the BT549 control groups (P < 0.01), which was then confirmed in WB experiments (Fig. 2B). In addition, we also successfully knocked down the CD147 expression in the MCF-7-liposome-si-CD147 group, which had significant lower CD147 level than MCF-7, MCF-7-liposome and MCF-7-liposome-siNC cell groups (P < 0.01) (Fig. 2C–D).

### CD147 impact on breast cancer cell migration and invasion

**Fig. 3:**
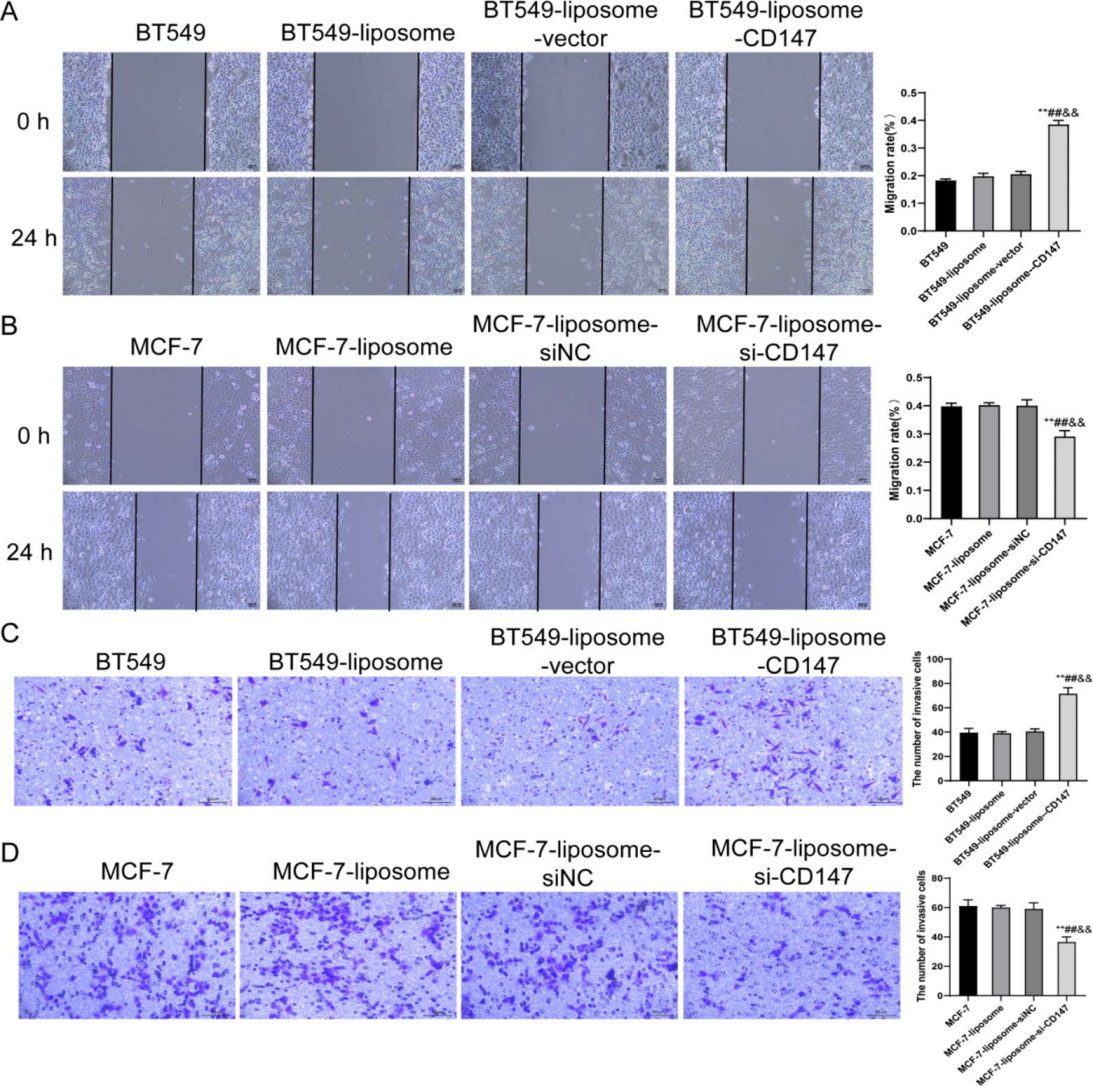
Effects of CD147 on cellular migration and invasion in BT549 and MCF-7 cells. (**A**) Scratch assay reveals enhanced migration in BT549-liposome-CD147. (**B**) Scratch assay shows reduced migration in MCF-7-liposome-si-CD147. (**C**) Transwell invasion assay for BT549 cell groups shows increased invasion in BT549-liposome-CD147. (**D**) Transwell invasion assay for MCF-7 cell groups displays reduced invasion in MCF-7-liposome-si-CD147. **P < 0.01, vs. BT549/MCF-7; ##P < 0.01, vs. BT549-liposome/MCF-7-liposome; &&P < 0.01, vs. BT549-liposome-CD147/MCF-7-liposome-siNC

In this experiment, we examined the migratory and invasive capabilities of BT549 and MCF-7 cell groups. Within the BT549 cell-based groups, no significant differences in invasion abilities were observed between the BT549, BT549-liposome and BT549-liposome-vector groups, while the BT549-liposome-CD147 group exhibited a significant increase in migratory capacity (P < 0.01) (Fig. 3A). Comparatively, the MCF-7-liposome-si-CD147 group displayed a significant decrease in migratory abilities compared to the MCF-7, MCF-7-liposome and MCF-7-liposome-siNC groups (P < 0.01) (Fig. 3B). Same as the migration capacity, the BT549-liposome-CD147 group showed a significant increase in the invasion capability of BT549 cells (P < 0.01) and the MCF-7-liposome-si-CD147 group was significantly reduced the invasive potential of MCF-7 cells (Fig. 3C–D). Overall, these findings indicate a potential role of CD147 in promoting cell migration and invasion in the BT549 and MCF-7 cell lines.

### CD147 enhanced the 5-FU resistance of breast cancer cells

**Fig. 4:**
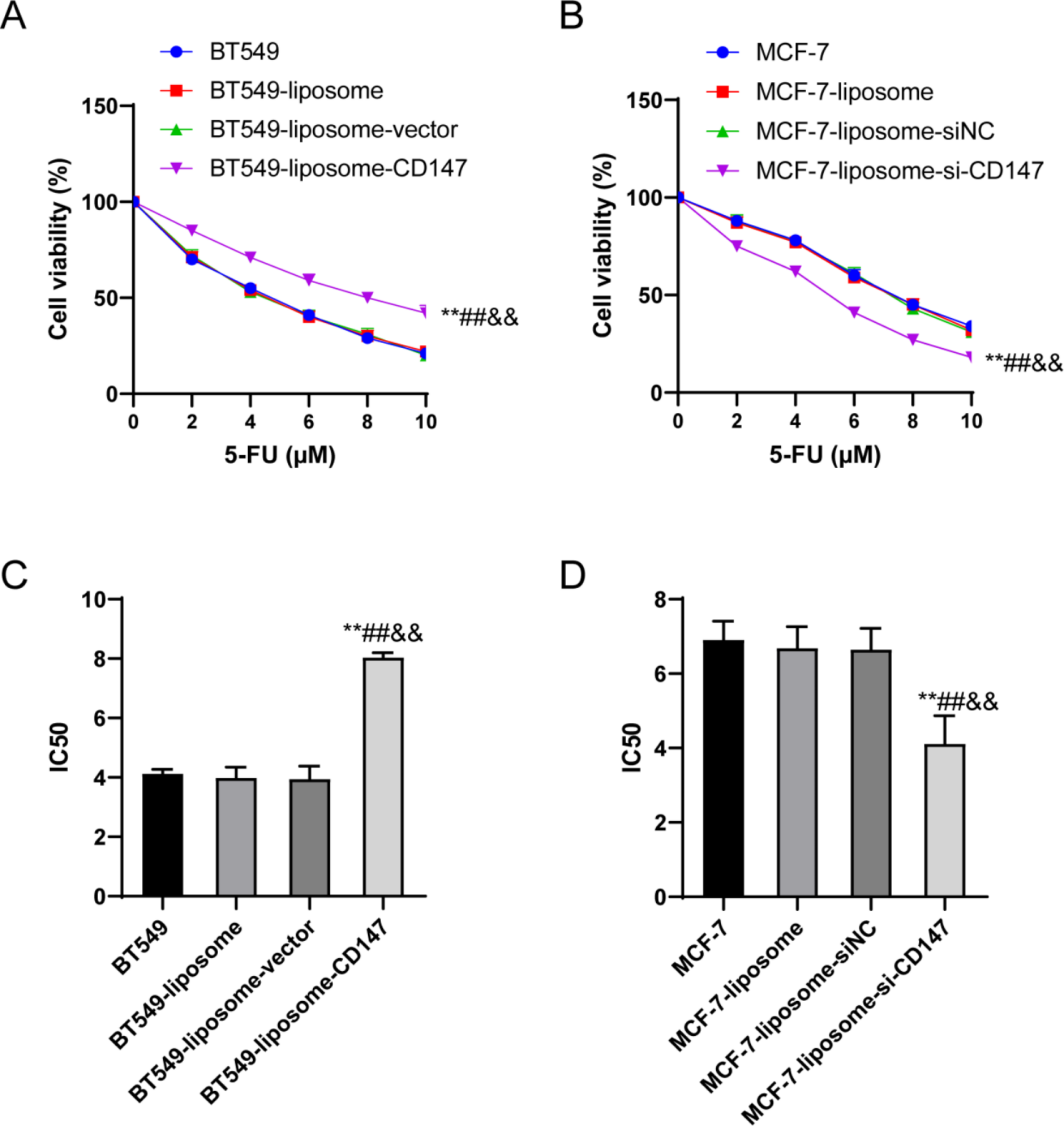
Effects of CD147 on 5-FU resistance in breast cancer cells. (**A**) CCK-8 assay indicates increased cell viability in BT549-liposome-CD147 at higher 5-FU concentrations. (**B**) CCK-8 assay demonstrates reduced cell viability in MCF-7-liposome-si-CD147 at higher 5-FU concentrations. (**C**) Increased IC50 value in BT549-liposome-CD147 cells. (**D**) Reduced IC50 value in MCF-7-liposome-si-CD147. **P < 0.01, vs. BT549/MCF-7; ##P < 0.01, vs. BT549-liposome/MCF-7-liposome; &&P < 0.01, vs. BT549-liposome-CD147/MCF-7-liposome-siNC.

The sensitivity of the different breast cancer cell lines to 5-FU was determined by evaluating the cells viability using CCK-8 assay in the BT549 and MCF-7 cell groups following treatment with different concentrations of 5-FU. In both BT549 cell groups and MCF-7 cell groups, there were no significant differences in cell viability at a 5-FU concentration of 0 µM among groups, when the 5-FU concentration increased from 2 µM to 10 µM, the cells viability gradually decreased (Fig. 4A–B). Notably, compared with the BT549 group, BT549-liposome group and BT549-liposome-vector group, the BT549-liposome-CD147 group displayed a significant increase in cell viability (P < 0.01) (Fig. 4A), while the MCF-7-liposome-si-CD147 group exhibited a significant lower cell viability than the MCF-7 group, MCF-7-liposome group and MCF-7-liposome-siNC group (P < 0.01) (Fig. 4B).

Furthermore, when assessing the half-maximal inhibitory concentration (IC50) for 5-FU, there were no significant differences among the BT549 group, BT549-liposome group, and BT549-liposome-vector group, but when compared with these groups, the IC50 value for BT549 cells in the BT549-liposome-CD147 group significantly increased (P < 0.01) (Fig. 4C). For the MCF-7 cells,, the IC50 value for MCF-7 cells in the MCF-7-liposome-si-CD147 group significantly decreased compared to the MCF-7 group, MCF-7-liposome group, and MCF-7-liposome-siNC group (P < 0.01) (Fig. 4D). These results indicated that CD147 could regulate the 5-FU resistance in breast cancer cells.

### CD147 induced EMT in breast cancer cells

**Fig. 5:**
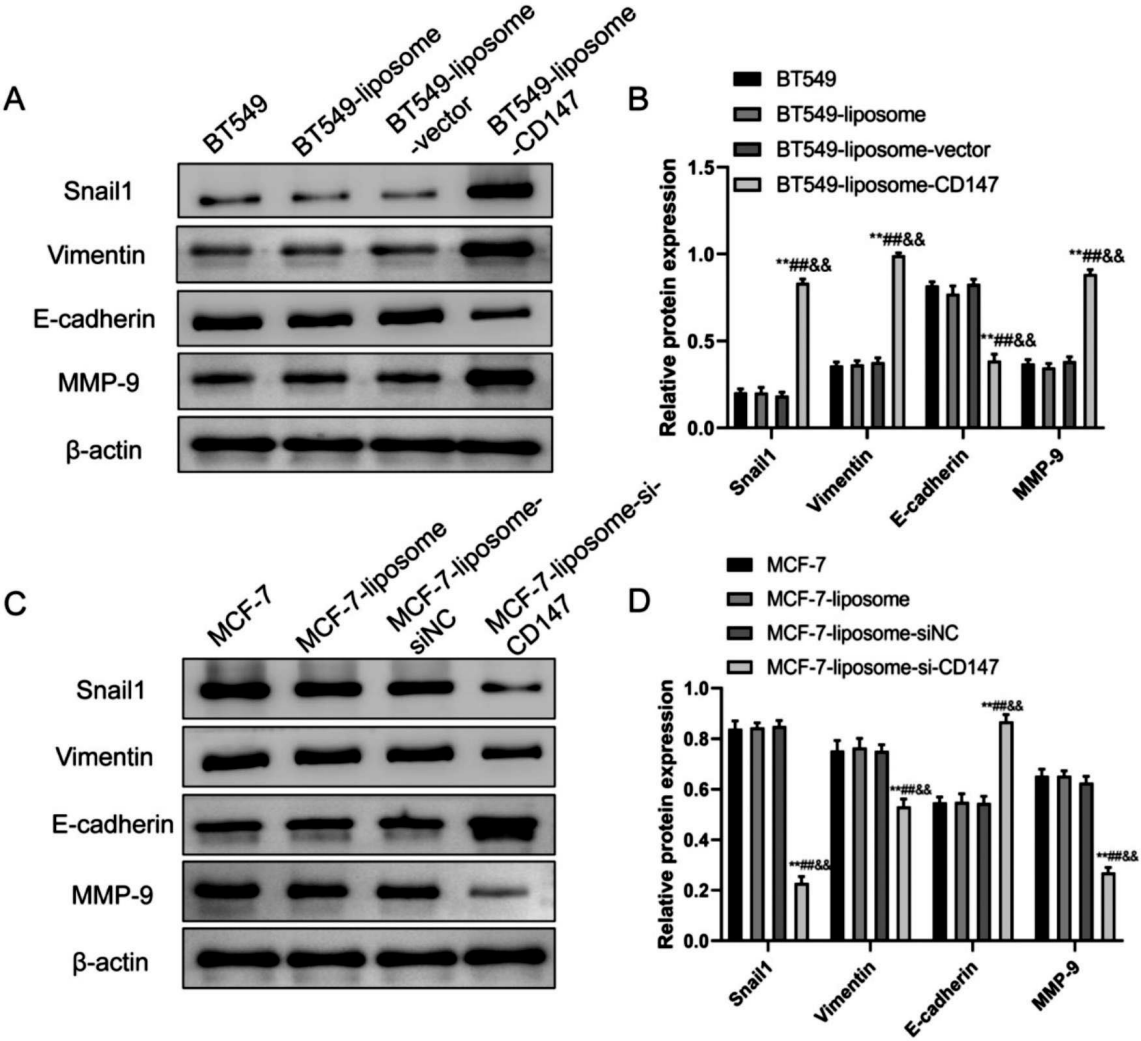
The expression of snail1, vimentin, E-cadherin and MMP-9 in BT549 and MCF-7 cells. (**A**–**B**) Western blot images (**A**) and semi-quantification (**B**) of Snail1, Vimentin, E-cadherin and MMP-9 proteins in BT549, BT549-liposome, BT549-liposome-vector and BT549-liposome-CD147. (C-D) Western blot images (**C**) and semi-quantification (**D**) of Snail1, Vimentin, E-cadherin and MMP-9 proteins in MCF-7, MCF-7-liposome, MCF-7-liposome-vector and MCF-7-liposome-siCD147 cell lines. Data were represented as the mean ± SD. **P < 0.01, vs. BT549/MCF-7; ##P < 0.01, vs. BT549-liposome/MCF-7-liposome; &&P < 0.01, vs. BT549-liposome-CD147/MCF-7-liposome-siNC

Here, we examined the expression of Vimentin, MMP-9 and E-cadherin and the impact of CD147 in different cell line variants of the BT549 and MCF-7 cells (Fig. 5). For the BT549, BT549-liposome and BT549-liposome-vector groups, The WB results showed no significant differences in Snail1, Vimentin, E-cadherin and MMP-9 expression levels, however, when compared to these groups, the BT549-liposome-CD147 group displayed a noteworthy increase in Snail1, Vimentin and MMP-9 expression as well as a significant decrease in E-cadherin (P < 0.01) (Fig. 5A–B). Similarly, for the MCF-7, MCF-7-liposome, and MCF-7-liposome-siNC groups, there were no significant differences in Snail1, Vimentin, E-cadherin and MMP-9 expression levels, while the MCF-7-liposome-si-CD147 group exhibited a significant increase in E-cadherin expression as well as a significant decrease in Snail1, Vimentin and MMP-9 expression (P < 0.01) (Fig. 5C–D). Collectively, both cell lines showed similar results within their respective groups, emphasizing the notable impact of CD147 on the expression of these critical proteins in cellular processes.

### CD147 activated the MAPK/ERK pathway in breast cancer cells

**Fig. 6:**
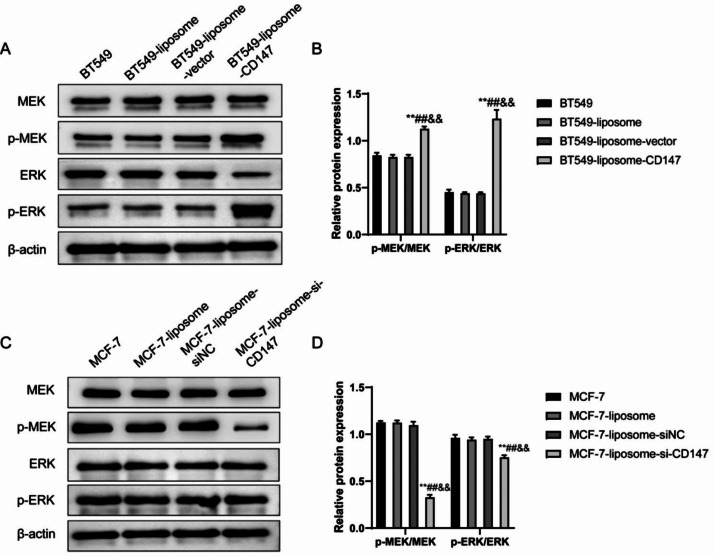
The expression of pERK and tERK in BT549 and MCF-7 cells. (**A**) Western blot analysis of MEK, p-MEK, ERK, and p-ERK protein levels in BT549 cell groups, indicating increased p-MEK and p-ERK in BT549-liposome-CD147. (**B**) Ratios of p-MEK/MEK and p-ERK/ERK significantly increase in BT549-liposome-CD147. (**C**) Western blot displays reduced p-MEK and p-ERK levels in MCF-7-liposome-si-CD147. (**D**) Decreased p-MEK/MEK and p-ERK/ERK ratios in MCF-7-liposome-si-CD147 indicate MAPK/ERK pathway modulation. **P < 0.01, vs. BT549/MCF-7; ##P < 0.01, vs. BT549-liposome/MCF-7-liposome; &&P < 0.01, vs. BT549-liposome-CD147/MCF-7-liposome-siNC

Among the BT549, BT549-liposome and BT549-liposome-vector, no significant differences were observed between them in regards to MEK, p-MEK, ERK and p-ERK protein levels, while the BT549-liposome-CD147 group, showed a significant increase in p-MEK/MEK ratio and p-ERK/ERK ratio (P < 0.01) (Fig. 6A–B). Conversely, the MCF-7-liposome-si-CD147 group showed a significant reduction in p-MEK/MEK ratio and p-ERK/ERK ratio compared with the MCF-7, MCF-7-liposome, and MCF-7-liposome-siNC groups (P < 0.01) (Fig. 6C–D). These findings indicated that CD147 activated the MAPK/ERK pathway both in BT549 and MCF-7 cell lines.

## Discussion

Drug resistance and tumor metastasis are the two important causes of treatment failure and mortality in cancer patients [22]. Tumor metastasis is a complex process involving multiple pathways and factors. EMT of the tumor cells increases their ability to migrate and invade nearby tissues, eventually resulting in the formation of new metastatic foci [23]. Several studies have shown that CD147 is closely involved in cancer metastasis [24–26]. The results of our study reveal significant insights into the role of CD147 in breast cancer cell behavior and its potential impact on various cellular processes. We initially observed high levels of CD147 expression in multiple breast cancer cell lines compared with normal breast epithelial cells, including BT549, MDA-MB-231 and MCF-7, with differential expression patterns among them, indicating its potential as a promising therapeutic target for breast cancer treatment. Additionally, the successful manipulation of CD147 levels in BT549 and MCF-7 cells demonstrates the feasibility of controlling CD147 expression, opening avenues for potential treatment strategies [27]. From a clinical standpoint, CD147’s differential expression among cell lines, especially its significant elevation in MCF-7 cells, hints at its relevance for prognosis and treatment response prediction. Investigating CD147 as a potential prognostic marker in breast cancer patient samples could provide valuable insights.

CD147 has also been implicated in chemoresistance, which is the main cause of treatment failure [28]. The choice of 5-FU in breast cancer cell lines for this study holds historical significance, reflects progress in cancer research, and offers valuable clinical implications. 5-FU is a chemotherapy drug with a long history of use in cancer treatment, including breast cancer [29]. It inhibits DNA synthesis, making it effective against rapidly dividing cancer cells [30]. Here, we intended to determine whether CD147 mediated the chemoresistance of metastatic breast cancer cells. We found that CD147 overexpression decreased the sensitivity of breast cancer cells to 5-FU. The reason may be that CD147 stimulates hyaluronic acid production and upregulates HER-2, which interacts with the cell surface receptor CD44 and indirectly induces multidrug resistance [31]. In terms of clinical implications, our findings regarding CD147’s role in mediating 5-FU resistance have potential relevance for breast cancer patients because understanding the molecular mechanisms behind resistance can inform treatment strategies and the development of combination therapies that target CD147 alongside 5-FU may enhance treatment efficacy and overcome resistance. Furthermore, these insights contribute to the ongoing evolution of breast cancer treatment, emphasizing the need for personalized, targeted approaches to improve patient outcomes.

EMT is a crucial process in cancer metastasis, allowing cancer cells to become more invasive and mobile, and has been closely related to poor prognosis [31, 32]. Therefore, studies are increasingly focusing on the regulatory pathways of EMT in tumor metastasis to identify novel therapeutic targets [33]. EMT is the process of epithelial cells into mesenchymal cells. During the process of EMT, the expression of marker proteins of epithelial cells (such as E-cadherin and Cytokeratin) is reduced, while the expression of marker proteins of mesenchymal cells (such as MMPs and Vimentin) is increased [34, 35]. Snail1 is an EMT regulator, belonging to the Snail superfamily of transcriptional repressors, which represses E-cadherin expression by binding to E-boxes in the promoter region of the proximal E-cadherin gene and induces EMT, plays a critical role in tumor metastasis [36]. In this study, the observed increase in Snail1, Vimentin and MMP-9 expression as well as the reduction in E-cadherin expression in the BT549-liposome-CD147 group suggests that CD147 may contribute to EMT and facilitate tumor invasiveness in certain breast cancer cases. Conversely, the reduction in Snail1, Vimentin and MMP-9 expression as well as the increase in E-cadherin expression in the MCF-7-liposome-si-CD147 group indicate that targeting CD147 could potentially reverse EMT in breast cancer cells. Overall, these findings imply that CD147 may serve as a potential therapeutic target for modulating the EMT behavior of breast cancer cells. In this regard, our findings were concordant with existing literature as CD147 has previously been shown to stimulate the production and secretion of MMPs [37] that facilitate tumor invasion and metastasis by breaking down the extracellular matrix [38].

Further research and clinical investigations are warranted to assess the feasibility of targeting CD147 in breast cancer treatment and its potential to impact patient outcomes.

Previous studies have shown that the MAPK/ERK pathway is involved in cancer metastasis and drug resistance [9, 39]. Kim et al. reported that CD147 induces the expression of MMP-9 in macrophages through ERK and NF-κB [40]. Our findings in regard to the MAPK/ERK pathway may have some credible clinical implications for breast cancer treatment. The activation of the MAPK/ERK pathway by CD147, as demonstrated in this study, suggests CD147 is a potentially considerable target for therapeutic intervention of breast cancer. Given that the MAPK/ERK pathway is known to be involved in cancer metastasis and drug resistance, inhibiting this pathway may hold promise for mitigating these critical aspects of breast cancer progression [41, 42]. Previous studies have revealed various different pathways by which CD147 promotes tumor metastasis and invasion. Zhang et al. reported that silencing of CD147 inhibited the migration and invasion of lung adenocarcinoma cells through blocking the Rap1 signaling pathway [24]. Dana et al’s data indicated that CD147 promotes the migration and invasion of cholangiocarcinoma cells by activating the Akt-FoxO3-NF-κB-MCT-1/4 axis [25]. The study of wang et al. demonstrated that CD147 promotes hepatocellular carcinoma cells collective invasion via upregulating cathepsin B expression [26]. It’s no doubt that CD147, as an extracellular matrix metalloproteinase inducer, can promote migration and invasion of the different tumors, but its mechanism is not fully confirmed. Our study’s results suggest that targeting CD147 could represent a novel approach to suppress the MAPK/ERK pathway and thereby potentially reduce the invasive behavior of breast cancer cells and inhibit EMT. Such therapeutic strategies, if validated in further studies and clinical trials, could offer new avenues for improving the management of breast cancer, particularly in cases where CD147-mediated pathway activation contributes to disease aggressiveness and resistance to standard treatments.

In future experiments, researchers could use preclinical studies in animal models to assess the therapeutic potential of CD147 inhibition in breast cancer. The successful manipulation of CD147 levels in BT549 and MCF-7 cells suggests promising treatment strategies, such as the development of targeted therapies, potentially through monoclonal antibodies or small molecule inhibitors aimed at reducing CD147 expression. RNA interference (RNAi) approaches could also be explored utilizing siRNA or other delivery methods. Furthermore, combination therapies involving CD147-targeted agents alongside standard chemotherapy drugs, immunotherapeutic strategies, and the initiation of clinical trials to evaluate the safety and efficacy of these approaches in breast cancer patients are all potential avenues for further investigation. These strategies aim to mitigate CD147-mediated effects, including drug resistance and enhanced invasive behavior, aiming to improve breast cancer treatment outcomes.

## Conclusion

In summary, our study provides compelling evidence for the significant influence of CD147 on breast cancer cell behavior, including invasion, migration, cell viability, drug resistance and EMT process. Additionally, our findings suggest a potential mechanistic link between CD147 and the MAPK/ERK pathway in mediating these effects. These insights contribute to a better understanding of CD147’s role in breast cancer progression and may hav e implications for the development of targeted therapies aimed at modulating CD147-related pathways in breast cancer treatment.

### Electronic supplementary material

Below is the link to the electronic supplementary material.

Supplementary Material 1: The full-length gels and blots of Western blotting in the article

## Data Availability

The datasets used and/or analysed during the current study available from the corresponding author on reasonable request.

## List of abbreviations

EMT: epithelial-mesenchymal transition
TNM: tumor-node-metastasis
VEGF: vascular endothelial growth factor
FCS: fetal calf serum
WB: Western blotting
TBST: Tris-buffer saline
ECL: enhanced chemiluminescence
CCK-8: Cell Counting Kit-8
SD: standard deviation
ANOVA: analysis of variance
IC50: inhibitory concentration
RNAi: RNA interference
