# Supplementary material for: CD147 promotes breast cancer migration and invasion by inducing epithelial-mesenchymal transition via the MAPK/ERK signaling pathway
Source: BMC Cancer. 2023 Dec 8;23:1214. doi: 10.1186/s12885-023-11724-2 (PMC10709944; doi:10.1186/s12885-023-11724-2)
Supplement: Supplementary file 1 — Supplementary Material 1: The full-length gels and blots of Western blotting in the article [file 12885_2023_11724_MOESM1_ESM.pdf]

Figure 1

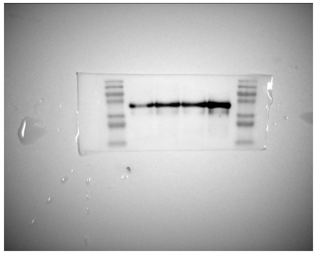

The original Western Blot images of CD147 in Figure 1. From left to right: MCF-10A, BT549, MDA-MB-231, and MCF-7.

Figure 1

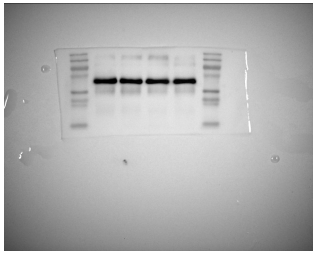

The original Western Blot images of β-actin in Figure 1. From left to right: MCF-10A, BT549, MDA-MB-231, and MCF-7.

Figure 2A

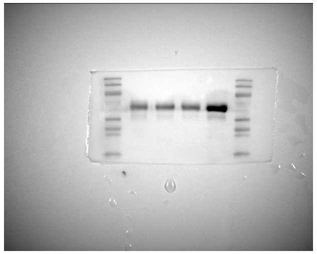

The original Western Blot images of CD147 in Figure 2A. From left to right: BT549, BT549-liposome, BT549-liposome-vector, BT549-liposome-CD147 .

Figure 2A

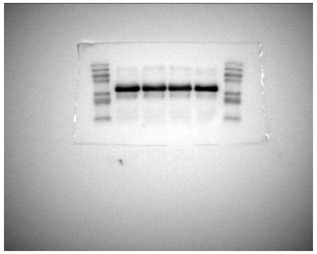

The original Western Blot images of β-actin in Figure 2A. From left to right: BT549, BT549-liposome, BT549-liposome-vector, BT549-liposome-CD147 .

Figure 2D

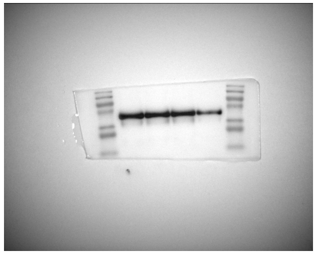

The original Western Blot images of CD147 in Figure 2D. From left to right: MCF-7, MCF-7-liposome, MCF-7-liposome-siNC, MCF-7-liposome-CD147 .

Figure 2D

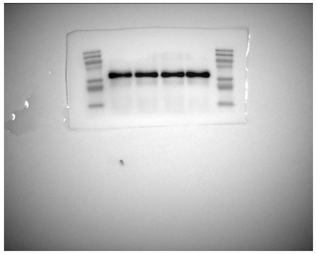

The original Western Blot images of β-actin in Figure 2D. From left to right:MCF-7, MCF-7-liposome, MCF-7-liposome-siNC, MCF-7-liposome-CD147 .

Figure 5A

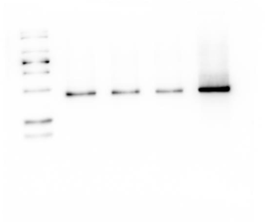

The original Western Blot images of Snail1 in Figure 5A. From left to right: BT549, BT549-liposome, BT549-liposome-vector, BT549-liposome-CD147 .

Figure 5A

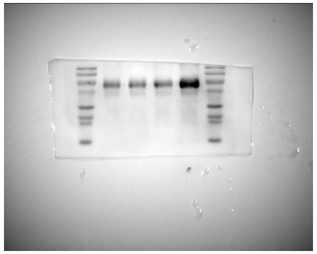

The original Western Blot images of Vimentin in Figure 5A. From left to right: BT549, BT549-liposome, BT549-liposome-vector, BT549-liposome-CD147 .

Figure 5A

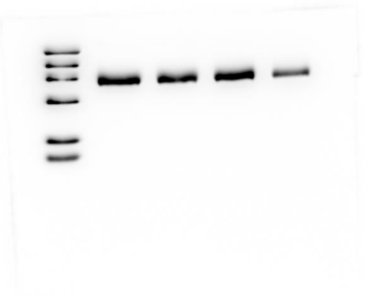

The original Western Blot images of E-cadherin in Figure 5A. From left to right: BT549, BT549-liposome, BT549-liposome-vector, BT549-liposome-CD147 .

Figure 5A

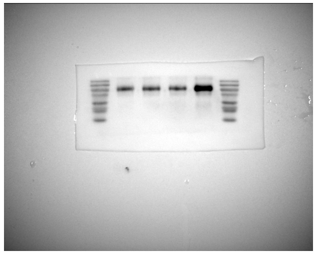

The original Western Blot images of MMP-9 in Figure 5A. From left to right: BT549, BT549-liposome, BT549-liposome-vector, BT549-liposome-CD147 .

Figure 5A

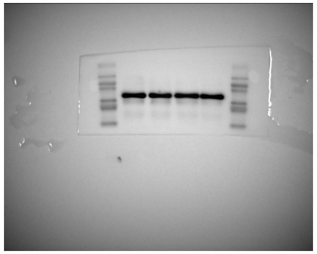

The original Western Blot images of β-actin in Figure 5A. From left to right: BT549, BT549-liposome, BT549-liposome-vector, BT549-liposome-CD147 .

Figure 5C

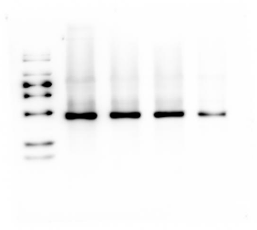

The original Western Blot images of Snail1 in Figure 5C. From left to right: MCF-7, MCF-7-liposome, MCF-7-liposome-siNC, MCF-7-liposome-CD147 .

Figure 5C

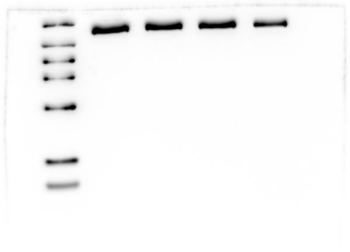

The original Western Blot images of Vementin in Figure 5C. From left to right: MCF-7, MCF-7-liposome, MCF-7-liposome-siNC, MCF-7-liposome-CD147 .

Figure 5C

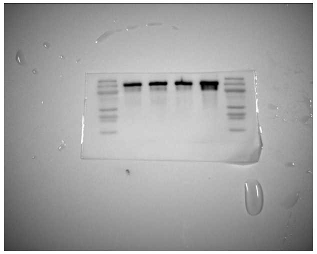

The original Western Blot images of E-cadherin in Figure 5C. From left to right: MCF-7, MCF-7-liposome, MCF-7-liposome-siNC, MCF-7-liposome-CD147 .

Figure 5C

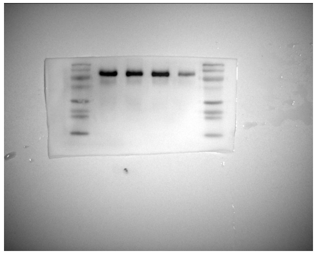

The original Western Blot images of MMP-9 in Figure 5C. From left to right: MCF-7, MCF-7-liposome, MCF-7-liposome-siNC, MCF-7-liposome-CD147 .

Figure 5C

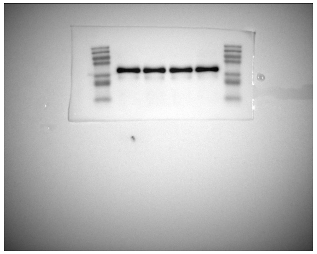

The original Western Blot images of β-actin in Figure 5C. From left to right:MCF-7, MCF-7-liposome, MCF-7-liposome-siNC, MCF-7-liposome-CD147 .

Figure 6A

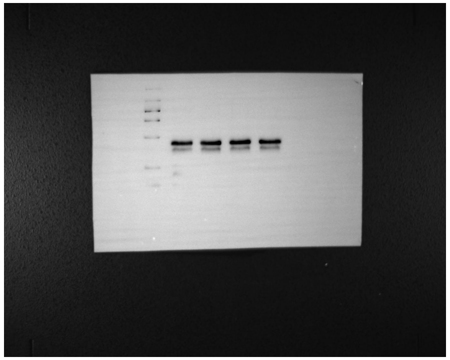

The original Western Blot images of MEK in Figure 6A. From left to right: BT549, BT549-liposome, BT549-liposome-vector, BT549-liposome-CD147 .

Figure 6A

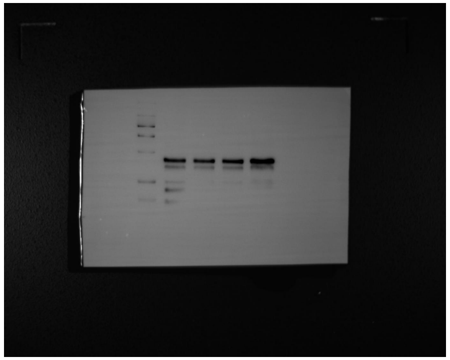

The original Western Blot images of P-MEK in Figure 6A. From left to right: BT549, BT549-liposome, BT549-liposome-vector, BT549-liposome-CD147 .

Figure 6A

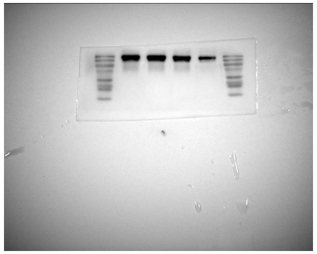

The original Western Blot images of ERK in Figure 6A. From left to right: BT549, BT549-liposome, BT549-liposome-vector, BT549-liposome-CD147 .

Figure 6A

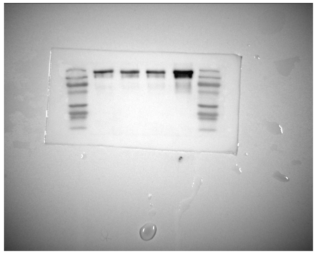

The original Western Blot images of P-ERK in Figure 6A. From left to right: BT549, BT549-liposome, BT549-liposome-vector, BT549-liposome-CD147 .

Figure 6A

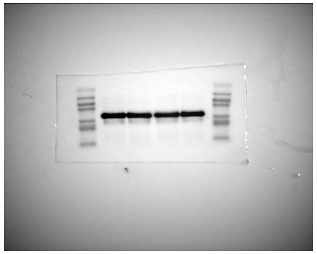

The original Western Blot images of β-actin in Figure 6A. From left to right: BT549, BT549-liposome, BT549-liposome-vector, BT549-liposome-CD147 .

Figure 6C

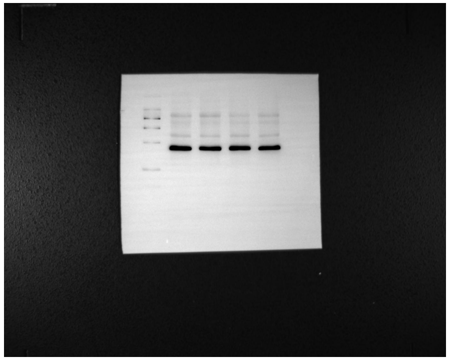

The original Western Blot images of MEK in Figure 6C. From left to right:MCF-7, MCF-7-liposome, MCF-7-liposome-siNC, MCF-7-liposome-CD147 .

Figure 6C

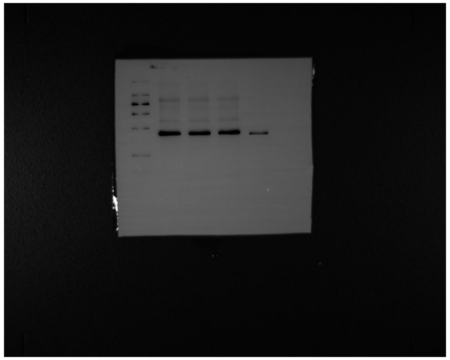

The original Western Blot images of P-MEK in Figure 6C. From left to right:MCF-7, MCF-7-liposome, MCF-7-liposome-siNC, MCF-7-liposome-CD147 .

Figure 6C

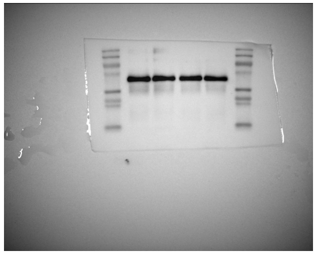

The original Western Blot images of ERK in Figure 6C. From left to right:MCF-7, MCF-7-liposome, MCF-7-liposome-siNC, MCF-7-liposome-CD147 .

Figure 6C

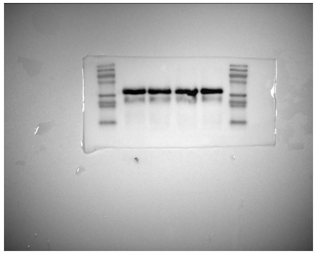

The original Western Blot images of P-ERK in Figure 6C. From left to right:MCF-7, MCF-7-liposome, MCF-7-liposome-siNC, MCF-7-liposome-CD147 .

Figure 6C

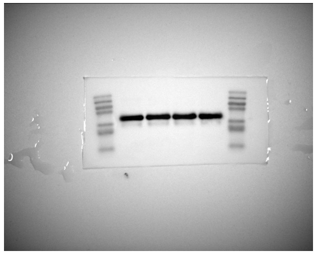

The original Western Blot images of β-actin in Figure 6C. From left to right:MCF-7, MCF-7-liposome, MCF-7-liposome-siNC, MCF-7-liposome-CD147 .
